# Epidemiology of Kawasaki Disease, Its Incomplete Form and Outcomes: A Single-Institution Experience in the United Arab Emirates

**DOI:** 10.7759/cureus.51320

**Published:** 2023-12-30

**Authors:** Abdulqader AL Zubaidi, Ghassan Ghatasheh, Venkatachalam Karuppaswamy, Hassib Narchi

**Affiliations:** 1 Department of Academic Affairs, Tawam Hospital, Al Ain, ARE; 2 Department of Pediatric Infectious Diseases, Tawam Hospital, Al Ain, ARE; 3 Department of Pediatric Cardiology, Tawam Hospital, Al Ain, ARE; 4 Pediatrics, United Arab Emirates University, Al Ain, ARE

**Keywords:** coronary artery aneurysm, complications, infants, cardiac sequelae, atypical kawasaki disease, mucocutaneous lymph node syndrome, kawasaki disease (kd)

## Abstract

Background and objective

Kawasaki disease is a childhood vasculitis, the leading cause of acquired heart disease in children worldwide. Data is lacking in the United Arab Emirates and the Middle East region. We aimed to review the clinical characteristics of patients diagnosed with Kawasaki disease, the response to intravenous immunoglobulin, and the short-term and long-term echocardiographic findings.

Study design

This is a retrospective cohort study involving patients diagnosed with Kawasaki disease in Tawam Hospital from January 2011 to December 2021.

Results

A total of 74 patients with a mean age of 36 months were diagnosed with Kawasaki disease, of whom 18 (24%) were below one year of age. Complete Kawasaki disease criteria were fulfilled in 36 patients (49%), while the remaining 38 (51%) were incomplete Kawasaki disease. A positive response to intravenous immunoglobulin occurred in less than 36 hours in 60 patients (84.5%). Echocardiography at the diagnosis of Kawasaki disease was performed on 71 patients, of whom 18 (25.35%) had cardiac involvement. The odds of coronary artery aneurysms in Kawasaki disease were 0.12 at diagnosis. Patients younger than 12 months were significantly more likely to be diagnosed with incomplete Kawasaki disease as compared to older patients (24 vs. 14 patients,* P *= 0.01). In the multivariate logistic regression analysis, only corticosteroid therapy remained statistically significantly associated with the development of coronary aneurysms (adjusted odds ratio (OR) 13.02, ci 1.05, 161.18; *P *= 0.045).

Conclusion

A high proportion of patients with Kawasaki disease had an atypical presentation, especially when under one year of age. There was no association between clinical characteristics or laboratory findings and prompt response to treatment within 36 hours.

## Introduction

Kawasaki disease, also known as mucocutaneous lymph node syndrome, is one of the acute vasculitis of childhood. It was originally described as a distinct clinical entity in Japanese pediatric patients by Dr. Tomisaku Kawasaki in 1967 and was first mentioned in English literature in 1974. Kawasaki disease may lead to coronary artery aneurysms in up to 25% of untreated cases. It has been reported worldwide and in developed countries as the leading cause of acquired heart disease in children [1-4].

An improvement in the cardiac outcome has been reported in patients with Kawasaki disease in Japan from 6% in 2000 to 2.8% in 2012, linked to advancement in management during the acute phase, including administration of intravenous immunoglobulins and aspirin therapy. However, a recent study has described an increase in the proportion of non-responders to Intravenous Immunoglobulins and cardiac complications between 2015 and 2018 [5,6].

Aside from limited case reports, there is no published data from the United Arab Emirates about Kawasaki disease [7]. We aimed to review our experience with Kawasaki disease over the course of the study by analyzing clinical presentation, response to intravenous immunoglobulins therapy, and the development of short-term and long-term cardiac echocardiographic abnormalities.

## Materials and methods

A retrospective review of the electronic medical records for children diagnosed with Kawasaki disease in Tawam Hospital over 11 years (January 2011 to December 2021). International Classification of Diseases (ICD)-10 code M30.3 was used in the study. The diagnosis of Kawasaki disease is based on the American Heart Association (AHA) diagnostic criteria for complete Kawasaki disease and incomplete Kawasaki disease [4]. Criteria for the diagnosis are fever for five days, with four or more out of the following five major clinical findings: polymorphic rash, non-purulent conjunctivitis, cervical lymph node enlargement ≥ 1.5 cm, changes of the extremities, and changes in the oral mucosa. Patients with less than four of these criteria were labeled as having incomplete Kawasaki disease when other diagnoses were excluded.

Demographic and clinical data included age at diagnosis and gender, symptoms and physical findings at presentation, duration of fever, the time of intravenous immunoglobulin treatment, response to treatment, and laboratory findings were reviewed. Failure to respond to intravenous immunoglobulin was defined as persistent or recrudescent fever ≥36 hours after the initial intravenous immunoglobulin infusion was completed.

Echocardiography

A routine 2-dimensional echocardiographic evaluation was performed for cardiac function and structures with coronary arteries. Coronary artery aneurysms were defined according to the AHA as having a diameter Z score of ≥ 2.5. The vessel was considered ectasia when the coronary artery was larger than normal (dilated) and without a segmental aneurysm. Echocardiography was repeated within two weeks of the onset of illness, four to six weeks, and six to 12 months after that, depending on the initial findings.

Statistical analysis

Results were expressed as numbers (percentages), mean ± standard deviation for continuous variables with a normal distribution, and median (interquartile range) for continuous variables with a skewed distribution. For the univariate analysis of the association of the variables with the outcomes, we compared the categorical data with the Chi-square test, or otherwise, the Fisher's for small samples. A multivariate logistic regression model was constructed, using only the variables associated with the outcome in the univariate analysis with P<0.1. We reported the adjusted odds ratio (aOR) with 95% confidence intervals (CI). A two-sided P-value < 0.05 was considered statistically significant for all the analyses.

## Results

A total of 74 patients were diagnosed with Kawasaki disease over the 11-year period. The mean age was 36 months (range three months to 11 years), of whom 18 patients (24%) were below one year of age at diagnosis. Boys were more commonly affected (62%) than girls. 

The criteria for complete Kawasaki disease were fulfilled in 36 children (49%), while the remaining 38 (51%) were diagnosed with incomplete Kawasaki disease. Patients younger than 12 months were significantly more likely to be diagnosed with incomplete Kawasaki disease as compared to older children (24 vs. 14 patients, P= 0.01). The mean duration of fever until the diagnosis of Kawasaki disease was six days. The predominant clinical findings at the time of diagnosis were polymorphic rash and mucosal changes, present in 85% and 82% of the patients, respectively (Table 1). Only two (2.7%) patients developed Kawasaki shock syndrome. Three had a sterile retropharyngeal abscess, and one had aseptic meningitis.

Laboratory findings (Table 1) included anemia and hypoalbuminemia in 62% and 65% of children, respectively. Elevated alanine aminotransferase (ALT) was found in 43% of the patients, and 42% had thrombocytosis (≥450 x 10^9^/L).

**Table 1:** Clinical and laboratory characteristics of 74 Patients diagnosed with Kawasaki disease Results are expressed as number (percent), mean ± standard deviation, or median (interquartile range).

Clinical and laboratory characteristics	
Males	46 (62.16)
Age (months)	36 ± 30
Under one year of age	18 (24.32)
Origin	
Gulf Cooperation Council (GCC)	61 (82.4)
Non-Gulf Cooperation Council Arabs	6 (8.11)
South-East Asians	5 (6.76)
Caucasians	2 (2.70)
Kawasaki Disease type:	
Complete	36 (48.65)
Incomplete	38 (51.35)
Duration of fever (days) prior to diagnosis	6 [5.0, 8.0]
Non-purulent conjunctivitis	56 (75.68)
Cervical lymphadenopathy	33 (44.59)
Polymorphic rash	63 (85.14)
Mucosal involvement	61 (82.43)
Extremity swelling or desquamation	38 (51.35)
Diarrhea	22 (29.73)
Vomiting	23 (31.08)
Joint involvement	11 (14.86)
BCG scar reactivation	3 (4.05)
Gallbladder hydrops	5 (6.76)
Shock	2 (2.70)
White blood cell count (x 10^9^/L)	12.7 [8.0, 17.3]
Platelet count (x 10^9^/L)	411 [279.0, 542.0]
Hemoglobin concentration (g/L)	103 [93.0, 114.0]
Erythrocyte sedimentation rate (mm/hr)	114 [55.0, 173.0]
Serum C-reactive protein (mg/L)	51 ± 37
Serum sodium concentration (mmol/L)	135 ± 3
Serum albumin concentration (g/L)	28 ± 8
Serum alanine transaminase concentration (U/L)	26 [16.0, 64.0]
Sterile pyuria	19 (32.76)

Out of the 74 Patients diagnosed, only one (1.4%) was not treated as parents refused treatment and two patients (2.7%) were treated only with aspirin as the diagnosis was made late in the clinic visit after the acute phase. All remaining patients were administered intravenous immunoglobulin (2 grams per kilogram over 12 hours), 60 (93.2%) of whom also received aspirin. Two other patients (2.7%) received an alternative to aspirin (clopidogrel or dipyridamole) either because of Glucose-6-Phosphatase Dehydrogenase (G6PD) deficiency or because they were found to be influenza positive.

A positive response to intravenous immunoglobulin occurred in less than 36 hours in 60 patients (84.5%), while 11 (15.4%) patients showed recrudescence of fever and receiving a second dose of intravenous immunoglobulin, with another eight patients also receiving corticosteroid therapy (11.3%). The corticosteroid therapy was 2 milligrams per kilogram of intravenous methylprednisolone over five days followed by oral tapering over the following weeks.

**Figure 1:**
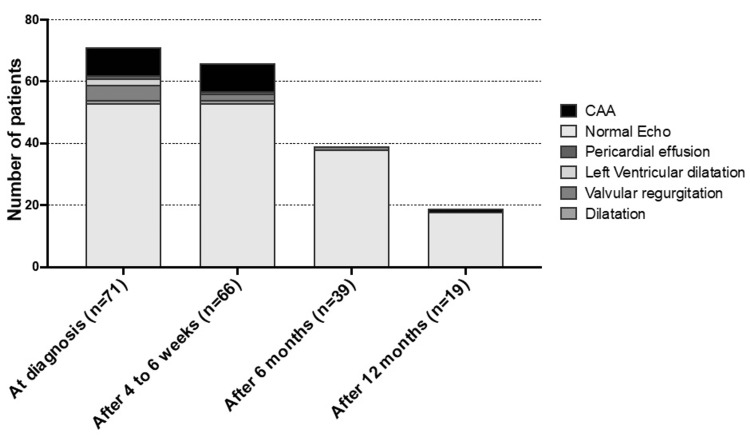
Echocardiographic findings in 71 children with Kawasaki disease CAA: Coronary artery aneurysm

Echocardiography at the diagnosis of Kawasaki disease was performed on 71 patients, of whom 18 (25.4%) had a cardiac involvement: nine patients had coronary artery aneurysm, one had coronary artery dilatation, five had valvular regurgitations, two had left ventricular dysfunction, and one had pericardial effusion. The mean duration of follow-up was 9 ± 11 months. Eight patients had persisting aneurysms at four to six weeks follow-up and one patient had a persistent coronary artery aneurysm after one year (Figure 1). The odds of coronary artery aneurysms in patients diagnosed with Kawasaki disease were 0.12 at diagnosis and 0.055 after 12 months of treatment (Table 2).

**Table 2:** Odds of coronary artery aneurysms in 71 patients with Kawasaki disease

Odds of coronary artery aneurysms	
	Odds (95% Confidence Interval)
At diagnosis	0.127 (0.086, 0.265)
After 4 to 6 weeks	0.138 (0.06. 0.28)
After 12 months	0.055 (0.007, 0.416)

Univariate and multivariate logistic regression analysis between clinical characteristics and laboratory findings, with response to treatment within 36 hours, showed no statistically significant association (Table 3). Univariate analysis for factors associated with coronary artery aneurysms showed a statistically significant association with age less than one year (Odds Ratio 7.08, Confidence Interval 1.48, 33.8; P= 0.014), cervical lymphadenopathy (Odds Ratio 0.14, Confidence Interval 0.016, 1.19, P= 0.072), elevated platelets count (Odds Ratio 1.0, Confidence Interval 1.000, 1.006; P= 0.016), corticosteroid therapy (Odds Ratio 4.91, Confidence Interval 0.74, 32.67; P=0.01). However, in the multivariate logistic regression analysis, adjusting for all variables, only corticosteroid therapy remained statistically significant (adjusted Odds Ratio 13.02, Confidence Interval 1.05, 161.18; P= 0.045) (Table 4).

**Table 3:** Factors associated with good response to treatment in < 36 hours in 74 patients with Kawasaki disease * Multivariate logistic regression on factors with a P value < 0.1 in the univariate analysis; Odds Ratio could not be computed for rash, bacillus Calmette-Guérin (BCG) scar reactivation, shock, or pyuria as they predicted the response perfectly; § Only for patients who did not respond to the first dose of intravenous immunoglobulin; NA not applicable.

Factors	Univariate model		Multivariate model	
	Odds Ratio (95% Confidence Interval)	P value	Odds Ratio (95% Confidence Interval)	P value*
Males	0.86 (0.23, 3.20)	0.826	NA	NA
Under one year of age	1.73 (0.34, 8.79)	0.503	NA	NA
Origin	0.58 (0.24, 1.37)	0.216	NA	NA
Kawasaki Disease type	1.14 (0.33. 3.94)	0.833	NA	NA
Duration of fever prior to diagnosis	1.14 (0.86, 1.49)	0.347	NA	NA
Non-purulent conjunctivitis	1.00 (0.23, 4.18)	1.00	NA	NA
Cervical lymphadenopathy	1.22 (0.35, 4.29)	0.751	NA	NA
Mucosal involvement	0.36 (0.04, 3.09)	0.355	NA	NA
Extremity swelling or desquamation	1.00 (0.29, 3.45)	1.00	NA	NA
Diarrhea	4.66 (0.54, 39.95)	0.160	NA	NA
Vomiting	1.26 (0.29, 5.41)	0.755	NA	NA
Joint involvement	0.34 (0.06, 1.85)	0.215	NA	NA
Gallbladder hydrops	0.57 (0.04, 8.05)	0.678	NA	NA
White blood cell count	1.13 (0.98, 1.30)	0.093	1.09 (0.90, 1.34)	0.347
Platelet count	1.00 (0.99, 1.00)	0.178	NA	NA
Hemoglobin concentration	1.01 (0.97, 1.04)	0.467	1.02 (0.99, 1.06)	0.099
Erythrocyte sedimentation rate	1.00 (0.99, 1.01)	0.447	NA	NA
C-reactive protein concentration	1.03 (1.00, 1.07)	0.036	NA	NA
Serum sodium concentration	1.07 (0.85, 1.35)	0.554	NA	NA
Serum albumin concentration	1.15 (0.98, 1.34)	0.072	1.14 (0.98, 1.34)	0.086
Serum alanine transferase concentration	1.00 (0.99, 1.02)	0.470	NA	NA
Corticosteroid therapy ^§^	NA	NA	NA	NA
Second dose of Intravenous Immunoglobulin ^§^	NA	NA	NA	NA
Coronary aneurysm at diagnosis	0.74 (0.13, 4.01)	0.728	NA	NA

**Table 4:** Factors associated with coronary artery aneurysm at diagnosis in 71 patients with Kawasaki disease * Multivariate logistic regression on factors with a P value < 0.1 in the univariate analysis; Odds Ratio could not be computed for rash, mucosal involvement, bacillus Calmette-Guérin (BCG) scar reactivation, shock, pyuria, gallbladder hydrops as they predicted the response perfectly.

Factors	Univariate model		Multivariate model*	
	Odds Ratio (95% Confidence Interval)	P value	Odds Ratio (95% Confidence Interval)	P value
Gender	0.53 (0.12, 2.35)	0.41	NA	NA
Under one year of age	7.08 (1.48, 33.8)	0.014	3.01 (0.49, 18.3)	0.232
Origin	1.77 (0.66, 18.78)	0.141	NA	NA
Kawasaki Disease type	3.57 (0.42, 6.45)	0.468	NA	NA
Duration of fever prior to diagnosis	1.16 (0.97, 1.40)	0.102	NA	NA
Non-purulent conjunctivitis	2.38 (0.27, 20.88)	0.433	NA	NA
Cervical lymphadenopathy	0.14 (0.016, 1.19)	0.072	0.27 (0.023, 3.21)	0.303
Extremity swelling or desquamation	0.48 (0.10, 2.18)	0.342	NA	NA
Diarrhea	0.94 (0.69, 1.29)	0.743	NA	NA
Vomiting	0.94 (0.66, 1.34)	0.759	NA	NA
Joint involvement	0.99 (0.97, 1.01)	0.473	NA	NA
White blood cell count	1.10 (0.97, 1.26)	0.128	NA	NA
Platelet count	1.00 (1.000, 1.006)	0.016	1.0 (0.99, 1.00)	0.052
Hemoglobin concentration	0.99 (0.95, 1.03)	0.701	NA	NA
Erythrocyte sedimentation rate	1.00 (0.99, 1.01)	0.680	NA	NA
C-reactive protein concentration	1.00 (0.98, 1.02)	0.563	NA	NA
Serum sodium concentration	1.03 (0.80, 1.34)	0.781	NA	NA
Serum albumin concentration	0.92 (0.78, 1.07)	0.308	NA	NA
Serum alanine transferase concentration	0.99 (0.98, 1.01)	0.528	NA	NA
Corticosteroid therapy	4.91 (0.74, 32.67)	0.01	13.02 (1.05, 161.18)	0.045
Second dose of Intravenous Immunoglobulin	0.85 (0.09, 7.82)	0.891	NA	NA

## Discussion

The epidemiological findings in our study show the mean age for Kawasaki disease is 3 years [4,8], and a higher incidence of Kawasaki disease is observed in males than in females, consistent with previously published studies [4,9,10].

The rate of cases of incomplete Kawasaki disease in our study (51%) was higher than in other previous studies where it ranged from 18-35% [11-14]. We also found a statistically higher incidence of incomplete Kawasaki disease in patients under one year of age, similar to reports from Italy (68.7%) and India (88%) [10,14]. The high incidence of incomplete Kawasaki disease in patients below one year of age highlights difficulties related to the atypical presentation and paucity of clinical signs at this young age. Therefore, a high index of suspicion is needed for infants under one year of age who present with fever without all suggestive symptoms of complete Kawasaki disease [10-15].

The proportion of cardiac abnormalities (25%) was less than in previous studies from Jordan, Switzerland, and Turkey, where it ranged between 38-65% [13,16,17]. Coronary artery aneurysms developed in 12.7% of patients, similar to two multicenter prospective studies published in France and the Netherlands where the incidence of coronary artery aneurysms was 14.3% and 13.5% respectively, but less than what was reported from Jordan, Algeria, and Turkey (20-41%) [9,13,17-19].

Previously published studies reported that young infants with Kawasaki disease are more likely to have cardiac sequelae compared to older patients. However, it remains unclear whether this is related to delay in diagnosis and management, or to other factors [4,10-14,20]. Although we found incomplete Kawasaki disease to be significantly more prevalent in patients below one year of age, we did not have a higher prevalence of cardiac complications. This may be attributed to the early diagnosis and treatment, as the mean duration of fever was only six days, and treatment with intravenous immunoglobulin in the acute phase and within 10 days is known to be effective in reducing the risk of coronary artery aneurysm [4,10,20].

Predictive risk factors for the development of coronary artery aneurysm in some reports included male gender, delay of treatment beyond 10 days, refractoriness to Intravenous Immunoglobulin, young age, and atypical presentations [9,13,19,21-23]. However, we found no significant clinical characteristics or laboratory findings associated with coronary artery aneurysm developments in the multivariant analysis adjusting for all explanatory factors, except for corticosteroid therapy which had a statistically significant association with coronary artery aneurysm. A possible explanation is that refractory Kawasaki disease, associated with cardiac complications, required corticosteroid therapy in our population.

A quick response to the initial intravenous immunoglobulin administration was observed in 84.5% of our patients, and a second administration of intravenous immunoglobulin was required in 15.4%, on whom 11.3% also received corticosteroid therapy, consistent with what has been reported by the AHA that 10-20% reported to have persistent or recrudescent of fever after initial treatment of Kawasaki disease with intravenous immunoglobulin. Moreover, recently published articles have reported persistence or recrudescence of fever ranging between 13-16%, and the steroid was given in 5.5% and 7% from data published from the Netherlands and Switzerland, respectively [4,9,12,16,17,19].

Limitations of our study include its retrospective nature, as complete data may be lacking in the patient's medical record. The echocardiographic follow-up was not systematic, and many patients missed appointments. It was a single-center study, and for that, the results may not be generalizable.

## Conclusions

A high proportion of patients with Kawasaki disease had an atypical presentation, especially when under one year of age. A high index of suspicion for Kawasaki disease is required in febrile patients, especially when under the age of one year. Further prospective multicenter studies are warranted in order to identify factors associated with a high prevalence of incomplete Kawasaki disease. The lower risk of coronary artery aneurysm in our study needs confirmation in a larger multi-center study to identify associated risk factors. Future studies should include a systematic and complete echocardiographic follow-up of affected patients.
